# Reduced T-Cell stemness underlies Th17 expansion and graft dysfunction in kidney transplant recipients

**DOI:** 10.3389/fgene.2025.1588941

**Published:** 2025-06-13

**Authors:** Chang Liu, Hao Jiang, Andu Zhu, Chen Xu, Zhenfan Wang, Guocai Mao, Minjun Jiang, Jianchun Chen, Zheng Ma, Jiaqian Qi, Zhijun Cao

**Affiliations:** ^1^ Department of Urology, Suzhou Ninth People’s Hospital, Soochow University, Suzhou, China; ^2^ Department of Urology, The First Affiliated Hospital of Soochow University, Suzhou, China; ^3^ Department of Clinical Laboratory, Suzhou Ninth People’s Hospital, Soochow University, Suzhou, China; ^4^ Department of Thoracic Surgery, Suzhou Dushu Lake Hospital, Dushu Lake Hospital Affiliated to Soochow University, Medical Centre of Soochow University, Suzhou, China; ^5^ Department of Thoracic Surgery, The First Affiliated Hospital of Soochow University, Soochow University, Suzhou, Jiangsu, China; ^6^ Department of Hematology, The First Affiliated Hospital of Soochow University, Suzhou, China

**Keywords:** T-cell stemness, Th17 cells, S100 proteins, kidney transplantation, immune regulation

## Abstract

**Introduction:**

End-stage renal disease (ESRD) is increasing worldwide, and although kidney transplantation improves survival, long-term graft loss–driven mainly by immune-mediated rejection–remains common. We aimed to delineate immune mechanisms that distinguish recipients with stable versus impaired graft function.

**Methods:**

Peripheral blood mononuclear cells from kidney-transplant recipients with normal (n = 10) or impaired (n = 10) renal function were profiled by single-cell RNA sequencing. Fourteen immune populations were identified; CD4^+^ T-cell “stemness” was quantified using mRNAsi and EREG_mRNAsi indices, lineage trajectories were reconstructed with Monocle, and ligand–receptor communication was inferred with iTalk. Findings were validated in an independent bulk RNA-seq cohort (n = 192) using differential expression and weighted gene co-expression network analysis (WGCNA).

**Results:**

Recipients with graft dysfunction exhibited (i) expansion of Th17 cells and contraction of Treg cells, (ii) significant loss of CD4^+^ T-cell stem-like features (lower mRNAsi/EREG_mRNAsi, p < 0.001), and (iii) pseudotime trajectories skewed toward Th17 differentiation. iTalk revealed enhanced S100A8/A9-TLR4 signalling from myeloid cells to neutrophils, consistent with reduced circulating neutrophils and presumptive intragraft accumulation. Bulk validation confirmed the stemness deficit and identified eight hub genes (API5, CAPRIN1, CCT2, DLG1, NMD3, RDX, SENP7, S100A4) that correlated with both low stemness and poor clinical outcome. Pathway enrichment implicated cell-morphogenesis, tight-junction, and metabolic-homeostasis pathways in graft injury.

**Discussion:**

Integrative single-cell and bulk analyses link diminished CD4^+^ T-cell stemness, Th17-dominant polarization, and S100A4-mediated neutrophil recruitment to graft dysfunction. These signatures nominate stemness indices, Th17/Treg balance, and the S100-TLR4 axis as candidate biomarkers and therapeutic targets to preserve allograft integrity and prolong transplant survival.

## Introduction

End-stage renal disease (ESRD) is a rapidly growing global health burden, with millions of patients worldwide requiring renal replacement therapy (Liyanage et al., 2015; Pippias et al., 2016; Robinson et al., 2016; Wu and Wu, 2018). Kidney transplantation is the definitive treatment for ESRD, offering improved survival and quality of life compared to dialysis (Muduma et al., 2016; Nankivell and Chapman, 2006; Sellares et al., 2012). However, long-term graft survival remains challenging, as immune-mediated rejection is a leading cause of allograft loss despite modern immunosuppression (Chapman et al., 2005; Pascual et al., 2012). Both acute and chronic rejection involve complex interactions between innate and adaptive immunity, underscoring the need for precise immune regulation to achieve durable graft tolerance. This persistent burden of graft dysfunction highlights an urgent need to elucidate the immune mechanisms governing transplant outcomes and to identify novel biomarkers predictive of allograft survival.

The post-transplant immune response is primarily orchestrated by CD4^+ T cells, which can differentiate into subsets that either drive inflammation or promote tolerance. Notably, T helper 17 (Th17) cells have been implicated in allograft rejection due to their production of proinflammatory cytokines, whereas regulatory T cells (Tregs) are crucial for suppressing alloimmune responses and facilitating long-term graft acceptance (Rutman et al., 2022). The balance between Th17 and Treg cells is thought to dictate allograft fate: an elevated Th17/Treg ratio is associated with poor graft function and heightened rejection risk (Gould and Auchincloss, 1999; Vincent-Schneider et al., 2002). In parallel, innate immune cells such as neutrophils contribute to graft injury by exacerbating inflammation–they infiltrate the allograft early and can act as antigen-presenting cells that activate T cells, as well as release factors that amplify adaptive immune cascades (Scozzi et al., 2017). Despite the well-defined roles of these immune populations, their intercellular crosstalk and functional plasticity in transplant recipients with divergent outcomes remain incompletely understood (Azim et al., 2023).

Stemness, traditionally a hallmark of cancer and pluripotent cells, has recently gained traction in immunology as a determinant of cellular plasticity and self-renewal capacity. For example, a PD1^+^ TCF1^+^ CD4^+^ T cell population with stem-like properties was identified in a tumor setting, capable of self-renewal and generating diverse effector T cells (Cardenas et al., 2024). To quantify such stem-like characteristics, an mRNA expression-based stemness index (mRNAsi) was developed; higher mRNAsi scores reflect a cell’s gene expression similarity to embryonic stem cells (i.e., a less differentiated state) (Chen et al., 2022). An epigenetically regulated counterpart, EREG-mRNAsi, integrates DNA methylation features with transcriptomic data to more comprehensively gauge cell differentiation status (Malta et al., 2018). In the context of transplantation, we hypothesize that preserving CD4^+^ T cell “stemness” is important for maintaining immune homeostasis, whereas loss of these stem-like properties may bias cells toward terminal differentiation into pathogenic effectors such as Th17 cells. Despite the emerging links between immune stemness and disease progression in other fields, its contribution to kidney allograft outcomes remains largely unexplored, warranting a detailed investigation of stemness dynamics in transplant T cell populations.

While the opposing roles of Th17 and Treg cells in rejection versus tolerance are well established, the interplay between T cell stemness, immune cell networking, and long-term graft outcome is poorly defined. For instance, the cellular and molecular pathways underlying operational tolerance in kidney transplantation remain unclear (Azim et al., 2023). High-dimensional techniques like single-cell RNA sequencing now enable unprecedented resolution in profiling immune states, but few studies have integrated these approaches with stemness indices in transplant research. Moreover, existing studies lack an integrative framework that combines longitudinal differentiation analysis (pseudotime trajectory), intercellular communication modeling (e.g., iTalk), and network-driven gene co-expression analysis to unravel the mechanisms of immune dysregulation. Addressing these gaps is critical for identifying novel therapeutic targets to restore immune equilibrium and ultimately improve graft survival.

This study aims to comprehensively characterize the immune landscape of kidney transplant recipients with divergent graft outcomes, with a special focus on CD4^+^ T cell subsets and their stemness properties. Using single-cell RNA sequencing of peripheral immune cells, coupled with pseudotime trajectory mapping and computational cell–cell communication analysis (iTalk), we will delineate the transcriptional programs governing CD4^+^ T cell differentiation and their crosstalk with neutrophils and other immune subsets. In addition, we employ weighted gene co-expression network analysis (WGCNA) on bulk transcriptomic data to identify key stemness-associated gene modules, which are further validated in an independent GEO cohort. We hypothesize that graft dysfunction is linked to an increased Th17/Treg ratio alongside diminished CD4^+^ T cell stemness, reflecting a shift toward a proinflammatory immune state. We propose that S100-family alarmins (such as S100A8/A9) mediate inflammatory crosstalk that promotes neutrophil infiltration and tissue injury in the allograft (Wang S. et al., 2018).

## Methods

### Patient enrollment and sample collection

This prospective study enrolled 32 adult kidney-transplant recipients with institutional review-board approval. All patients received a calcineurin-inhibitor–based triple immunosuppressive regimen—tacrolimus (n = 27) or ciclosporin (n = 5) in combination with mycophenolate mofetil and low-dose prednisone (5 mg day^-1^). An additional mTOR inhibitor was administered to 5 patients (15.6%), with no significant difference in distribution between the stable-function and graft-dysfunction groups. Before biopsy, acute viral, bacterial, and fungal infections were ruled out by physical examination, complete blood count, C-reactive protein, and pathogen-specific PCR assays for CMV, BKV, EBV, and SARS-CoV-2. Two patients in the stable-function group had low-level CMV viraemia, which did not affect subsequent clustering analyses.

Approximately 10 mL of peripheral blood was collected into EDTA tubes, typically in the morning (fasting state), and processed within 2 h. Exclusion criteria included active infection, recent hospitalization, and significant autoimmune comorbidities. The studies involving humans were approved by Suzhou Ninth People’s Hospital. The studies were conducted in accordance with the local legislation and institutional requirements. The participants provided their written informed consent to participate in this study. All procedures complied with the Declaration of Helsinki and institutional ethics guidelines.

### PBMC isolation and sample pooling

Peripheral blood mononuclear cells (PBMCs) were isolated using Ficoll–Hypaque density gradient centrifugation at 400 g for 30 min at room temperature. The mononuclear layer was carefully aspirated, washed twice in phosphate-buffered saline (PBS), and assessed for viability via trypan blue exclusion. Cells exceeding 90% viability were counted and adjusted to a uniform concentration. For each group (control vs. renal insufficiency), ten individual samples were pooled in equal cell numbers to form two composite PBMC preparations, thereby reducing inter-individual variability while preserving representative immune profiles. The pooled cells were then immediately prepared for single-cell RNA sequencing.

### Single-cell RNA-sequencing (scRNA-seq) and library preparation

Pooled PBMCs were resuspended at ∼1 × 10^6^ cells/mL in PBS supplemented with 0.04% bovine serum albumin. Cells were loaded onto a Chromium Controller (10x Genomics) without additional enzymatic or mechanical dissociation. Single-cell gel bead emulsions were generated following the manufacturer’s protocols, incorporating barcoded primers for reverse transcription. After cDNA amplification, libraries were constructed via fragmentation, end repair, A-tailing, and adaptor ligation steps. Final libraries were quantified by Qubit and fragment size verified by Bioanalyzer. Paired-end sequencing (2 × 150 bp) was conducted on an Illumina NovaSeq 6,000 at an average depth of ∼50,000 reads/cell, ensuring comprehensive transcript coverage for subsequent analyses.

### Quality control and preprocessing of scRNA-seq data

Raw sequencing reads were processed with Cell Ranger (10x Genomics, v3.1) to perform demultiplexing, read alignment (using GRCh38 as the reference), and feature counting. Cells with fewer than 200 or more than 6,000 detected genes were excluded, as were those with mitochondrial transcript counts exceeding 15%. Potential doublets were removed using DoubletFinder (v2.0). Remaining cells were normalized using a log-based method (Seurat, v4.0), applying scaling factors to mitigate library size differences. Highly variable genes were identified for downstream clustering, ensuring that global cell-to-cell heterogeneity was captured while minimizing technical noise.

### Dimensionality reduction and clustering

Highly variable genes were identified in Seurat (v4.0) using the FindVariableFeatures function, retaining approximately 2000 genes for downstream analysis. The dataset was then centered and scaled before Principal Component Analysis (PCA), and the top principal components were projected onto a two-dimensional UMAP (Uniform Manifold Approximation and Projection) space for visualization. Clustering was performed using the Louvain algorithm at varying resolution parameters (0.2–1.2). A final resolution of 0.5 was selected after comparing cluster stability and biological relevance, resulting in 14 discrete clusters (labeled 0–13). Each cluster was subsequently annotated based on canonical immune markers (e.g., CD3D for T cells, LYZ for myeloid cells) and known transcriptional profiles, enabling identification of neutrophils, CD4^+^ T cells, Th17 cells, Treg cells, and other key immune subsets.

### Differential expression and functional enrichment analyses

Differential expression was conducted via Seurat’s Wilcoxon rank-sum test (v4.0), comparing clusters or cell subsets to uncover genes up- or downregulated in each condition (false-discovery rate <0.05). Significantly enriched pathways were then identified using Gene Set Enrichment Analysis (GSEA) and KEGG annotation to elucidate functional distinctions among clusters. For stemness profiling, the mRNA stemness index (mRNAsi) and its epigenetically regulated variant (EREG_mRNAsi) were computed with a machine-learning algorithm, quantifying transcriptomic similarity to embryonic stem cells. Correlations between these indices and clinical/phenotypic parameters were subsequently evaluated to assess their prognostic and mechanistic relevance.

### Pseudotime trajectory analysis

Pseudotime trajectories were constructed using Monocle (v2.20), which orders cells along a learned developmental timeline based on transcriptomic similarity. To define input features, we selected either highly variable genes detected in Seurat or differentially expressed genes (DEGs) specific to key clusters of interest. This gene set was then used to project CD4^+^ T, Th17, and Treg cells onto a low-dimensional manifold, capturing their lineage progression and developmental branches. Monocle’s principal graph construction identified branch points where cells diverged, and we visualized expression trends of lineage-specific markers (e.g., S100A4, CCL5) over pseudotime to highlight potential regulatory events associated with T-helper cell fate decisions.

### Cell–cell communication modeling

Cell–cell communication was inferred using the iTalk R package (v0.1.0), which integrates ligand and receptor expression profiles across major immune subsets. After extracting normalized expression data, iTalk filtered out low-abundance genes and identified candidate ligand–receptor pairs based on a minimum expression threshold. Pairs deemed statistically significant (FDR <0.05) or functionally relevant were retained, such as S100–TLR4 interactions implicated in neutrophil recruitment. Visualization of these putative signaling networks was performed via circular and chord diagrams, highlighting key axes of immunomodulation and pinpointing potential molecular conduits of cross-talk between T cells and myeloid cells.

### Bulk RNA-seq analysis for cohort validation

An independent cohort of 192 kidney transplant recipients was obtained from the GEO database (accession GSE147451). Reads were aligned to the GRCh38 reference genome using STAR (v2.7) and quantified with featureCounts (v1.6). Normalized gene expression matrices were then processed to calculate mRNAsi and EREG_mRNAsi scores for each sample, following the established machine-learning protocol. Clinical data (e.g., graft function status) were used to stratify patients into good vs. poor outcome groups. Differential expression analyses were performed using DESeq2 (v1.30), and Weighted Gene Co-expression Network Analysis (WGCNA, v1.69) was conducted to identify modules correlated with stemness indices. This approach validated single-cell findings within a larger population-level dataset. In the bulk RNA-seq cohort, graft dysfunction was defined *a priori* as (i) a ≥25% fall in estimated glomerular filtration rate (eGFR). Patients meeting neither criterion were classified as stable function.

### Identification and characterization of key gene sets

Differentially expressed genes (DEGs) from the “good” vs. “poor” outcome comparison were intersected with genes from the WGCNA module most strongly associated with stemness indices (mRNAsi/EREG_mRNAsi). The resulting candidate gene set was further explored using the STRING database (v11.0) to construct a Protein–Protein Interaction (PPI) network, elucidating potential functional interconnections. Subsequently, Gene Ontology (GO) and KEGG pathway enrichment analyses were performed (ClusterProfiler v3.18) to identify relevant biological processes and pathways. This integrative approach highlighted critical gene clusters potentially mediating immune dysregulation and tissue remodeling in graft dysfunction.

### Statistical and computational tools

All statistical analyses were conducted using R (v4.0.5) unless otherwise specified. Comparisons between two groups (e.g., cell type abundance) were typically made using non-parametric Wilcoxon rank-sum or paired t-tests, contingent upon data distribution. Multi-group comparisons employed Kruskal–Wallis or one-way ANOVA, followed by appropriate *post hoc* tests. Differentially expressed genes were identified using Wilcoxon tests (Seurat) or DESeq2’s negative binomial model, and false discovery rate (FDR) corrections were applied to control for multiple testing. Pseudotime trajectories were generated in Monocle (v2.20), cell–cell communication was inferred via iTalk (v0.1.0), network analysis relied on WGCNA (v1.69), and PPI mapping utilized STRING (v11.0). For all comparisons, p < 0.05 or q < 0.05 (where applicable) was considered statistically significant.

### Ethical approval and data availability

This study was conducted in accordance with the principles of the Declaration of Helsinki and approved by the Institutional Review Board of Suzhou Nineth People‘s Hospital. Written informed consent was obtained from all participants prior to sample collection. Single-cell RNA sequencing data have been deposited in the Gene Expression Omnibus (GEO), and the bulk RNA-seq validation dataset was retrieved from [GSE147451]. All custom scripts for data processing, analysis, and figure generation are available upon reasonable request to the corresponding author.

## Results

### Distinct immune profiles and reduced CD4^+^ T-cell stemness in kidney transplant recipients with impaired renal function

**FIGURE 1:**
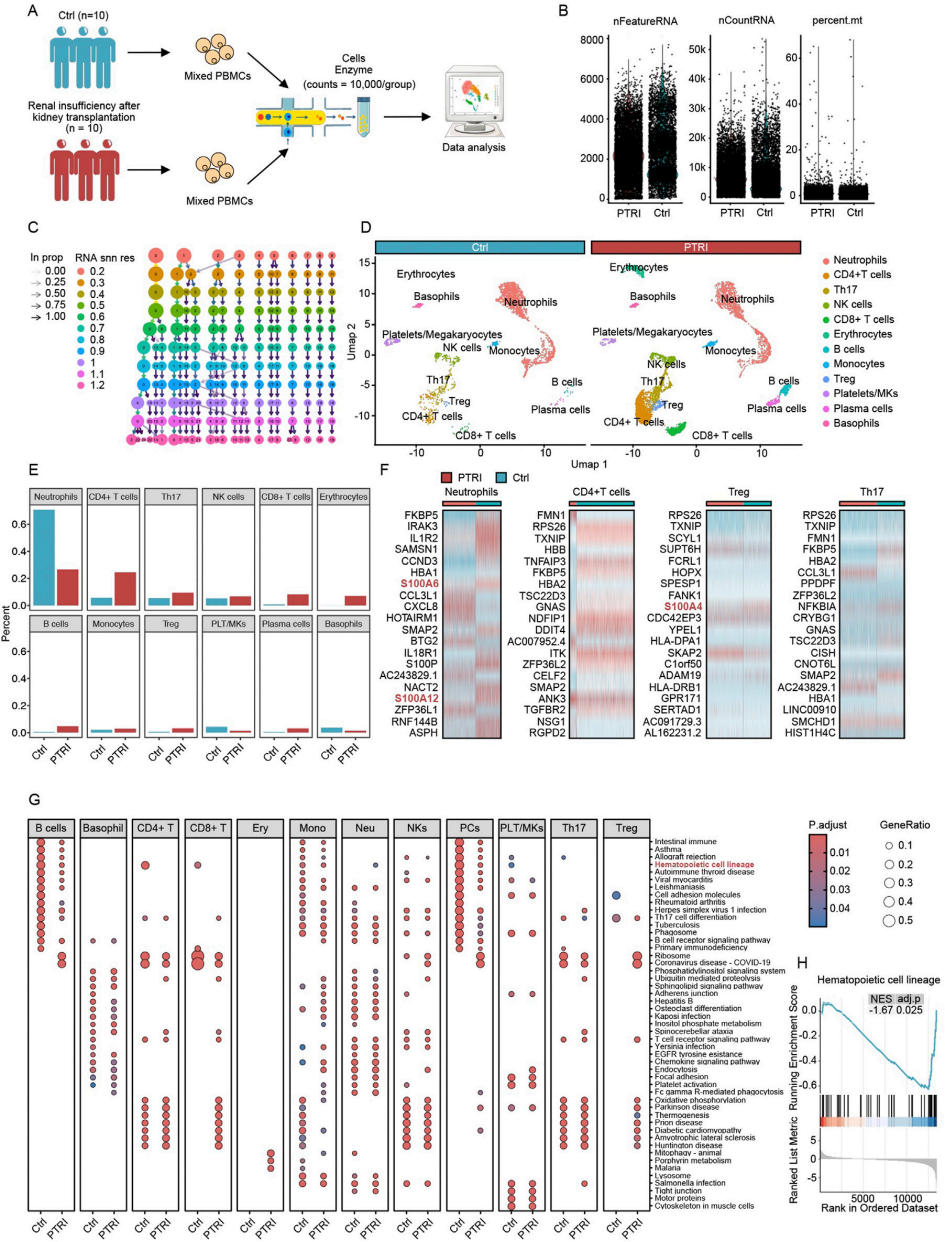
Single-cell transcriptomic profiling of PBMCs in kidney transplant recipients. **(A)** Schematic of the study design. PBMCs from 10 kidney transplant patients with normal renal function (Ctrl) and 10 with renal insufficiency (PTRI) were pooled into two groups. After enzymatic treatment and cell preparation, single-cell RNA-sequencing was performed, yielding over 10,000 cells per group for subsequent analyses. **(B)** QC metrics for both groups. Each dot represents a single cell, with three key parameters plotted: nFeatureRNA (number of detected genes), nCountRNA (total RNA count per cell), and percent. mt (percentage of mitochondrial reads). Both groups show similar distributions, indicating that the data passed QC and are suitable for further bioinformatic investigation. **(C)** Resolution testing across scRNA-seq data. Each column represents a clustering resolution from 0.2 to 1.2, and each node indicates a cluster identified at that resolution. Arrows show how clusters merge or subdivide as the resolution changes. Based on these findings, a resolution of 0.5 was chosen for downstream analysis. **(D)** UMAP visualization and cluster annotation. At resolution 0.5, 14 clusters (labeled 0–13) were defined according to established cell-type markers. The two panels compare the control (blue header) and renal insufficiency (red header) groups, revealing distinct immune cell populations such as neutrophils, CD4^+^ T cells, Th17 cells, Treg cells, and others. **(E)** Proportional comparison of major immune cell types in the control (blue bars) versus renal insufficiency (red bars) groups. Neutrophils are significantly reduced in the PTRI group, while CD4^+^ T cells, Th17 cells, and Treg cells are notably increased. **(F)** Heatmaps depicting key differentially expressed genes among neutrophils, CD4^+^ T cells, Treg cells, and Th17 cells. Several S100 protein family members (highlighted in red) show pronounced upregulation in the PTRI group, consistent with a heightened inflammatory or stress response. **(G)** Dot plots of KEGG pathway enrichment for each cell type in the Ctrl (left) and PTRI (right) groups. The color scale indicates adjusted p values, and the size of each circle corresponds to the gene ratio. Notably, CD4^+^ T cells in the PTRI group display diminished enrichment of stemness-related pathways, while neutrophils exhibit an enhanced stemness signature. **(H)** GSEA plot focusing on the “Hematopoietic cell lineage” pathway in CD4^+^ T cells. The negative enrichment score (NES = −1.67, adj. p = 0.025) in the PTRI group underscores a loss of stem-like features in this subset among patients with impaired renal function.

We collected peripheral blood from two groups of kidney transplant recipients: ten individuals with normal renal function (control group) and ten individuals with impaired renal function (renal insufficiency group). After pooling the PBMCs from each group, we performed single-cell RNA-sequencing (scRNA-seq) on these two mixed samples, acquiring over 10,000 high-quality cells per group. Figure 1A provides an overview of the study design, illustrating how each group’s PBMCs were pooled and prepared for scRNA-seq. To ensure the reliability of our data, we conducted rigorous quality control (QC) procedures. We excluded low-quality cells, doublets, and cells with abnormally high mitochondrial read percentages (percent.mt). As shown in Figure 1B, the control and renal insufficiency groups exhibit broadly comparable distributions for nFeatureRNA (total number of genes detected per cell), nCountRNA (total transcripts per cell), and percent.mt (fraction of mitochondrial reads). These QC metrics confirm that the datasets for both groups met the criteria required for downstream analyses.

Following quality control (QC) confirmation, we explored a range of clustering resolutions from 0.2 to 1.2 to determine the optimal parameter for identifying distinct immune cell populations within our single-cell dataset. As shown in Figure 1C, each resolution level yielded a different number and composition of clusters. Ultimately, we chose a resolution of 0.5 for our subsequent analyses, which produced 14 discrete clusters labeled from 0 to 13. We annotated these clusters based on canonical marker genes and visualized them using Uniform Manifold Approximation and Projection (UMAP), as illustrated in Figure 1D. Major cell types included neutrophils, CD4^+^ T cells, Th17 cells, regulatory T (Treg) cells, NK cells, CD8^+^ T cells, erythrocytes, B cells, monocytes, platelets/megakaryocytes, plasma cells, and basophils. Notably, the UMAP plots show that both control and renal insufficiency groups harbor comparable core immune subsets, although their relative abundances differ, as revealed in later analyses.

Building upon our annotated cell clusters, we next compared the relative abundance of each major immune population between the control (Ctrl) and renal insufficiency (PTRI) groups. As depicted in Figure 1E, neutrophils showed a marked decrease in the PTRI group, whereas CD4^+^ T cells, Th17 cells, and Treg cells exhibited significant increases. Notably, the magnitude of Th17 cell elevation exceeded that of Treg cells, suggesting a potentially heightened pro-inflammatory milieu in the PTRI group. To further investigate these findings, we conducted differential expression analyses on neutrophils, CD4^+^ T cells, Th17 cells, and Treg cells between the two groups. As shown in Figure 1F, multiple members of the S100 protein family (e.g., *S100A6*, *S100A12, S100A4*) were prominently upregulated in the PTRI group, indicating an enhanced inflammatory or stress-related response in these cell subsets. Consistent with the transcriptomic data, immunoblotting of PBMC lysates demonstrated a marked increase in S100A4 protein in recipients with graft dysfunction (PTRI) compared with clinically stable controls. When normalised to β-actin, densitometric analysis showed a ∼2.5-fold elevation in the PTRI group (mean ± SD: 2.63 ± 0.17 a. u. vs. 1.04 ± 0.06 a. u.; P = 0.008, two-tailed Wilcoxon test; Supplementary Figure S1A,B).

We performed KEGG pathway enrichment analyses across all identified cell subsets to compare functional differences between the control (Ctrl) and renal insufficiency (PTRI) groups. As shown in Figure 1G, several pathways displayed notable enrichment differences. Of particular interest, CD4^+^ T cells in the PTRI group exhibited a markedly reduced stemness potential, whereas neutrophils showed an enhanced stemness signature. To further explore the stemness-related changes in CD4^+^ T cells, we conducted a Gene Set Enrichment Analysis (GSEA). As illustrated in Figure 1H, the “Hematopoietic cell lineage” pathway was significantly negatively enriched in the PTRI group (NES = −1.67, adj. *p* = 0.025), suggesting a diminished regenerative or stem-like capacity in the CD4^+^ T cell subset of patients with renal insufficiency.

### Pseudotime analysis reveals divergent T-helper cell trajectories and elevated S100A4 in renal insufficiency

**FIGURE 2:**
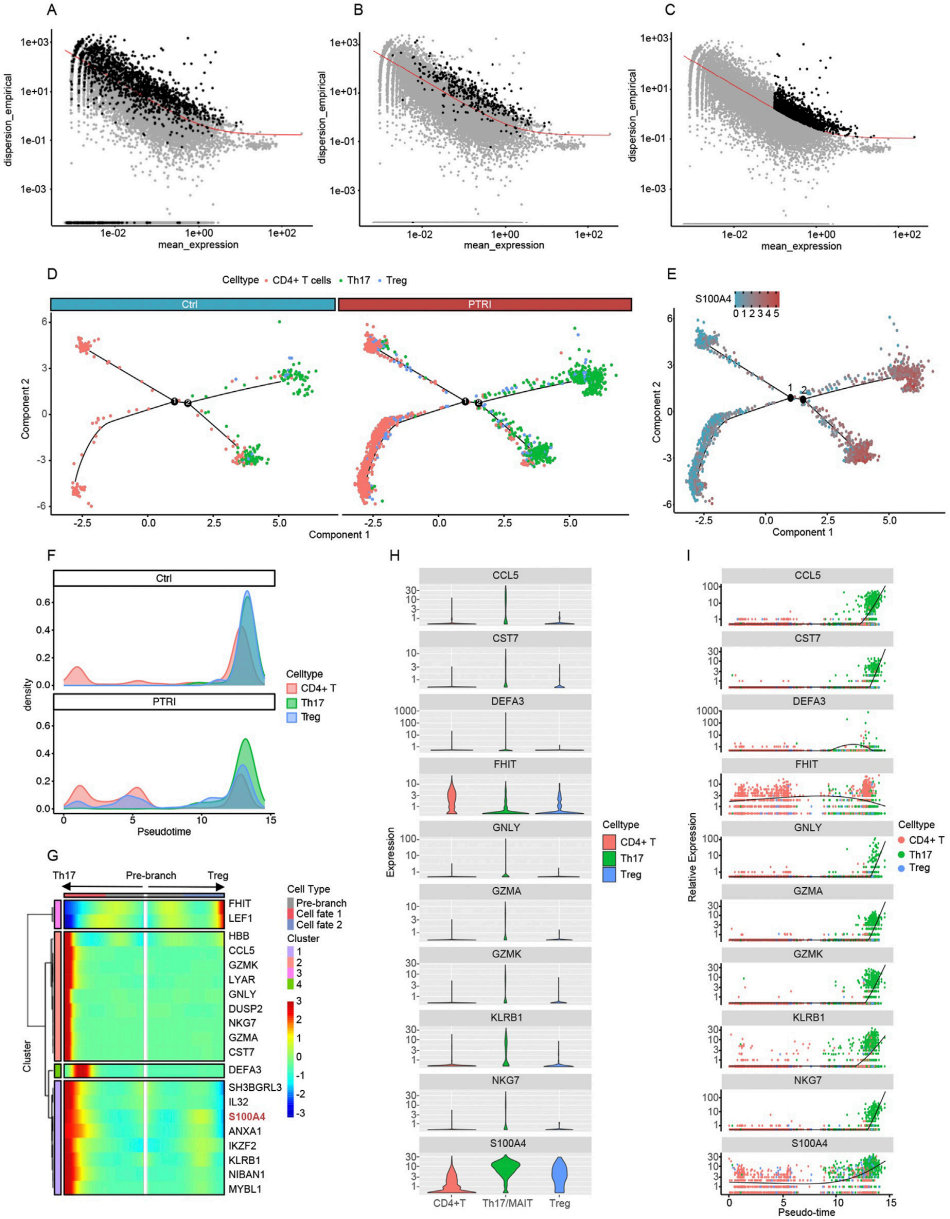
Gene selection and pseudotime analyses reveal divergent T-helper cell fates and elevated S100A4 expression. **(A)** Seurat-identified highly variable genes (HVGs). Each dot represents a gene, plotted by its mean_expression (x-axis) and empirical dispersion (y-axis). Black points above the red trend line indicate HVGs used in the pseudotime trajectory. **(B)** Cluster-based differentially expressed (DE) genes. Genes discovered through inter-cluster comparisons are highlighted in black, again illustrating their divergence from the expected dispersion trend. **(C)** Monocle-derived HVGs. Monocle’s native selection algorithm identifies a partially overlapping yet distinct set of HVGs (black points). These HVGs were likewise used for pseudotime construction of CD4^+^ T, Treg, and Th17 lineages. **(D)** Pseudotime trajectory of CD4^+^ T, Th17, and Treg cells in control (left) and PTRI (right) samples. Each branch point (black circles labeled “1” and “2”) represents a key bifurcation. CD4^+^ T cells (red) from the PTRI group show an increased differentiation toward Th17 cells (green) and a reduced transition into Tregs (blue), indicating diminished T-cell “stemness.” **(E)** Expression of S100A4 over the pseudotime trajectory. The heat-scale (blue to red) reflects S100A4 transcript levels across cells. Th17 cells exhibit notably higher S100A4 expression than CD4^+^ T or Treg cells, consistent with an enhanced pro-inflammatory state in the PTRI group. **(F)** Density plots illustrating pseudotime transitions among CD4^+^ T (red), Th17 (green), and Treg (blue) subsets. In the control group (top), cells predominantly converge on Treg cells, whereas in the renal insufficiency (PTRI) group (bottom), a larger fraction of cells progress toward the Th17 phenotype. **(G)** Heatmap of key genes regulating T-helper cell branching. The “pre-branch” region is flanked by two fates, Th17 (left) and Treg (right). Expression levels of S100A4 (highlighted in red) increase in the Th17 branch, suggesting a link between high S100A4 and the pro-inflammatory Th17 lineage. **(H)** Violin plots illustrating per-cell expression of the top 10 differentially expressed genes across CD4^+^ T (red), Th17 (green), and Treg (blue) subsets. The y-axis is shown on a logarithmic scale to accommodate wide expression ranges, with higher violin spread indicating greater gene expression. **(I)** Pseudotime trajectories highlighting changes in gene expression over the simulated developmental timeline. Each dot represents a single cell’s expression level, and the fitted line (black) indicates the overall trend. Notably, S100A4 expression surges with time and is highly enriched in Th17 cells, suggesting a potential pro-inflammatory role in these late-stage T-cell trajectories.

To investigate potential lineage relationships and dynamic transcriptional programs among CD4^+^ T cells, regulatory T (Treg) cells, and Th17 cells, we performed pseudotime trajectory analyses using three distinct strategies for identifying highly variable or differentially expressed genes. In Figure 2A, we show the empirical dispersion versus mean expression for genes selected by Seurat as highly variable (black points), whereas Figure 2B highlights the genes identified as differentially expressed among the clusters. Finally, Figure 2C depicts the set of highly variable genes determined by Monocle’s own selection algorithm. Each scatter plot illustrates the relationship between gene abundance (mean_expression on the x-axis) and dispersion_empirical (y-axis), with the red trend line representing the fitted dispersion model. Genes exceeding the model’s threshold (black points) were subsequently incorporated into a pseudotime analysis to reconstruct potential developmental trajectories of the T helper subsets. By comparing these three gene-selection approaches, we aimed to capture a comprehensive view of key regulators and transition markers that may underlie the functional plasticity and lineage specification of CD4^+^ T, Treg, and Th17 cells.

To further examine lineage commitment within CD4^+^ T-cell subsets, we constructed pseudotime trajectories that included CD4^+^ T cells, Th17 cells, and Treg cells for both control (Ctrl) and renal insufficiency (PTRI) groups. As illustrated in Figure 2D, patients in the PTRI group exhibited a reduced capacity for sustaining CD4^+^ T-cell “stemness,” instead displaying an increased propensity toward Th17 differentiation. Concurrently, the generation of Treg cells—known to modulate inflammation and mitigate allograft rejection—was comparatively diminished. These findings suggest that impaired renal function may skew T-helper cell fate toward a more pro-inflammatory profile. We next visualized the expression of *S100A4* along the pseudotime axis, focusing on key branch points for Th17 and Treg cells (Figure 2E). Notably, *S100A4* levels were substantially higher in Th17 cells than in CD4^+^ T or Treg subsets, mirroring our single-cell differential expression results. This elevated *S100A4* expression in Th17 cells further underscores the heightened inflammatory or pathogenic potential of the Th17 lineage within the renal insufficiency cohort.

To further delineate the temporal dynamics of CD4^+^ T, Th17, and Treg cell differentiation, we generated density plots (Figure 2F) and heatmaps (Figure 2G) from the pseudotime analysis. In the control group (top panel of Figure 2F), CD4^+^ T cells predominantly transition toward Treg cells as pseudotime progresses, suggesting a more robust capacity for anti-inflammatory regulation. In contrast, patients with impaired renal function (PTRI group, bottom panel of Figure 2F) exhibit a pronounced shift of CD4^+^ T cells into the Th17 fate, with fewer cells becoming Tregs. This skew toward Th17 lineage may contribute to the heightened pro-inflammatory milieu observed in renal insufficiency. Consistently, the heatmap in Figure 2G highlights genes associated with branching toward Th17 versus Treg cells. Notably, *S100A4* expression increases as cells commit to the Th17 lineage, in line with our previous findings that *S100A4* upregulation correlates with a pro-inflammatory phenotype. These patterns underscore a possible mechanism by which elevated *S100A4* may bias T-helper differentiation away from a regulatory fate, potentially exacerbating allograft injury in patients with renal insufficiency.

To further characterize genes driving the transition among CD4^+^ T, Th17, and Treg cells, we identified the top 10 differentially expressed genes along pseudotime. Figure 2H shows violin plots of these genes in each subset, demonstrating clear distinctions in expression patterns among CD4^+^ T (red), Th17 (green), and Treg (blue) cells. Figure 2I depicts how these same genes vary across pseudotime. Notably, *S100A4* levels rise progressively over the trajectory and peak in Th17 cells, reinforcing previous observations that link *S100A4* to a pro-inflammatory phenotype. Other genes, such as *CCL5* and *GNLY*, also show distinct temporal and subset-specific expression, underscoring their potential roles in T-cell lineage commitment and function.

To test whether the transcriptional shift toward a Th17-polarised CD4^+^ T-cell state is mirrored at the protein level, we quantified circulating Th17 cytokines in a subset of 15 kidney-transplant recipients. Plasma IL-17A concentrations were markedly higher in patients with poor graft funciton exhibited the high-Th17 signature than in recipients with a high-stemness profile associated with stable graft function (median 37 pg mL^-1^ vs. 16 pg mL^-1^; 2.3-fold difference; P = 0.004; Supplementary Figure S1C).

### Intercellular communication and reduced stemness in kidney transplant recipients with poor graft outcomes

**FIGURE 3:**
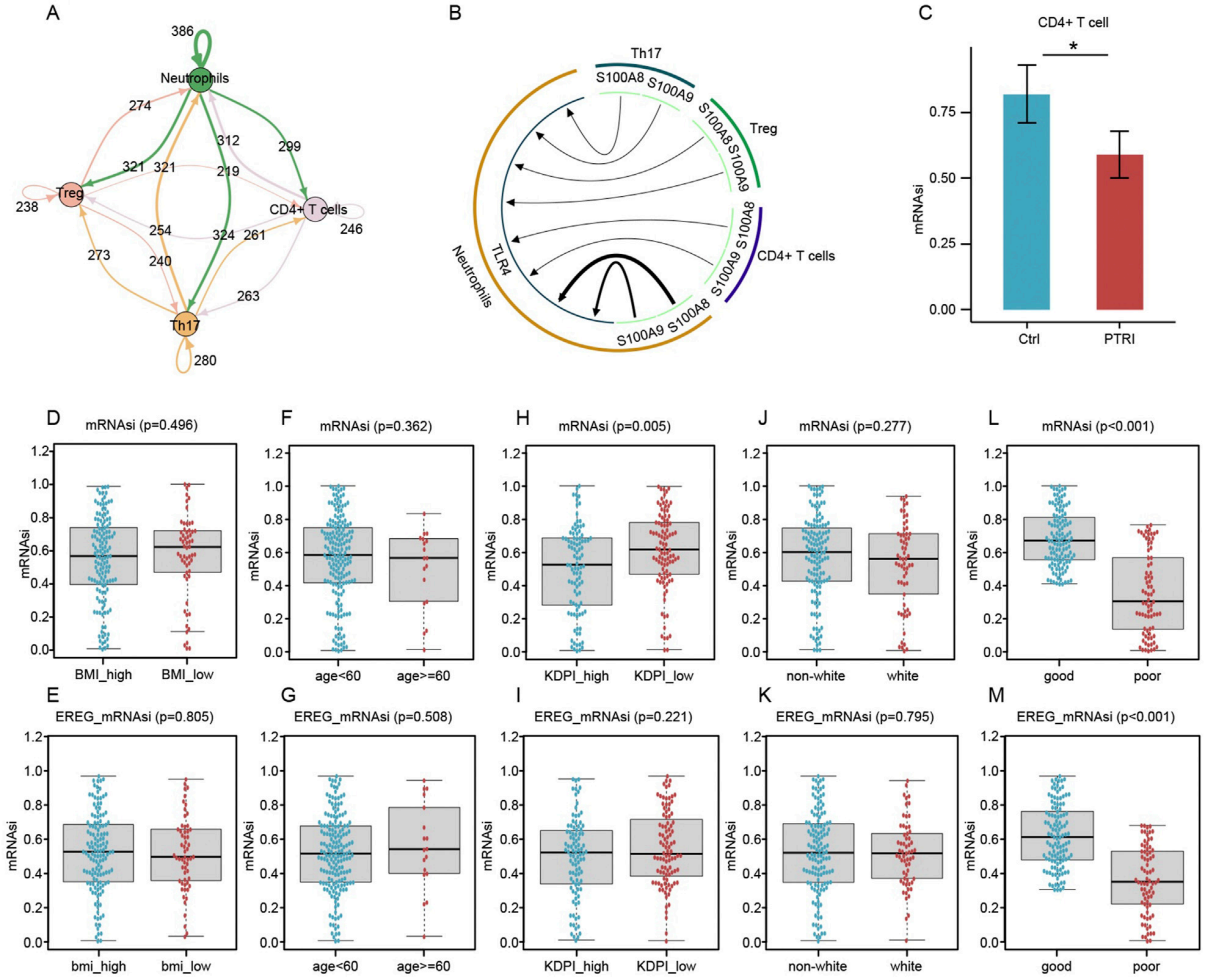
Intercellular communication, T-Cell stemness, and clinical outcome associations in kidney transplant recipients. **(A)** Network diagram illustrating the extensive interactions among Treg (pink node), Th17 (orange node), CD4^+^ T (purple node), and neutrophil (green node) subsets. Arrows indicate the direction of putative signaling, and numbers on each arrow represent detected ligand–receptor pairs. **(B)** Circular layout highlighting critical ligand–receptor relationships. S100 family ligands (S100A8, S100A9, etc.) (green arcs) bind to TLR4 on neutrophils (brown sector), potentially driving neutrophil recruitment to the graft site. Such infiltration may account for the observed decrease in circulating neutrophils in renal transplant recipients with impaired graft function. **(C)** Comparison of mRNA Stemness Index (mRNAsi) in CD4^+^ T Cells. Bar plots depict the mean ± SEM for mRNAsi values in CD4^+^ T cells isolated from control (blue) and PTRI (red) groups. The asterisk indicates a statistically significant difference (*p* < 0.05), consistent with reduced stemness in the PTRI subset. **(D–G)** Box-and-whisker plots contrasting mRNAsi **(D,F)** and EREG_mRNAsi **(E,G)** for subgroups defined by BMI (high vs. low) and age (<60 vs. ≥60). No significant differences were observed (p > 0.05). **(H,I)** mRNAsi **(H)** and EREG_mRNAsi **(I)** comparisons between high and low Kidney Donor Profile Index (KDPI) groups. Lower mRNAsi was noted in the KDPI_high group (p = 0.005), whereas EREG_mRNAsi did not reach statistical significance (p = 0.221). **(J,K)** Comparisons by race (non-white vs. white) did not reveal significant stemness differences (p > 0.05). **(L,M)** Patients classified as having “good” renal outcomes showed notably higher mRNAsi **(L)** and EREG_mRNAsi **(M)** than those with “poor” outcomes. The p < 0.001 result underscores a strong association between decreased stemness indices and impaired renal function post-transplant.

We performed intercellular communication analysis using the iTalk package, focusing on four immune cell subsets that exhibited the most pronounced proportional changes—CD4^+^ T cells, Th17 cells, Treg cells, and neutrophils. As shown in Figure 3A, the network diagram reveals extensive ligand–receptor interactions among these populations. Delving deeper into the specific pathways, we found that multiple S100 family proteins, including S100A8 and S100A9, can engage TLR4 on neutrophils (Figure 3B). This interaction may promote substantial neutrophil infiltration into the transplanted kidney tissue, potentially explaining why peripheral blood neutrophil counts are markedly reduced in patients with impaired graft function.

Given our earlier findings hinting at an aberrant “stemness” profile in the CD4^+^ T-cell population of patients with impaired renal function, we employed a machine learning algorithm to calculate the mRNA stemness index (mRNAsi). As illustrated in Figure 3C, CD4^+^ T cells from the renal insufficiency (PTRI) group displayed a significantly lower mRNAsi compared to those from the control (Ctrl) group (*p* < 0.05). These results suggest that the CD4^+^ T-cell subset in patients with impaired graft function may have reduced regenerative or multipotent capacity, potentially contributing to a skewed immune response post-transplant. To extend our findings to a larger patient cohort, we retrieved RNA-sequencing data from 192 kidney transplant recipients in the GEO database. We calculated the mRNA stemness index (mRNAsi) and its epigenetically regulated variant (EREG_mRNAsi) for each sample. As illustrated in Figures 3D–M, patients who subsequently developed impaired graft function (“poor” outcome) showed significantly lower stemness scores compared to those with stable renal function (“good” outcome). Notably, while clinical factors such as body mass index (BMI) or age did not correlate strongly with mRNAsi or EREG_mRNAsi (p > 0.05 in most comparisons), the difference in stemness indices between good and poor outcome groups was highly significant (p < 0.001). These results reinforce our earlier single-cell findings and suggest that diminished mRNAsi/EREG_mRNAsi may reflect a compromised regenerative capacity in the transplanted kidney.

### Differential gene expression and co-expression network analysis reveal a stemness-associated module

**FIGURE 4:**
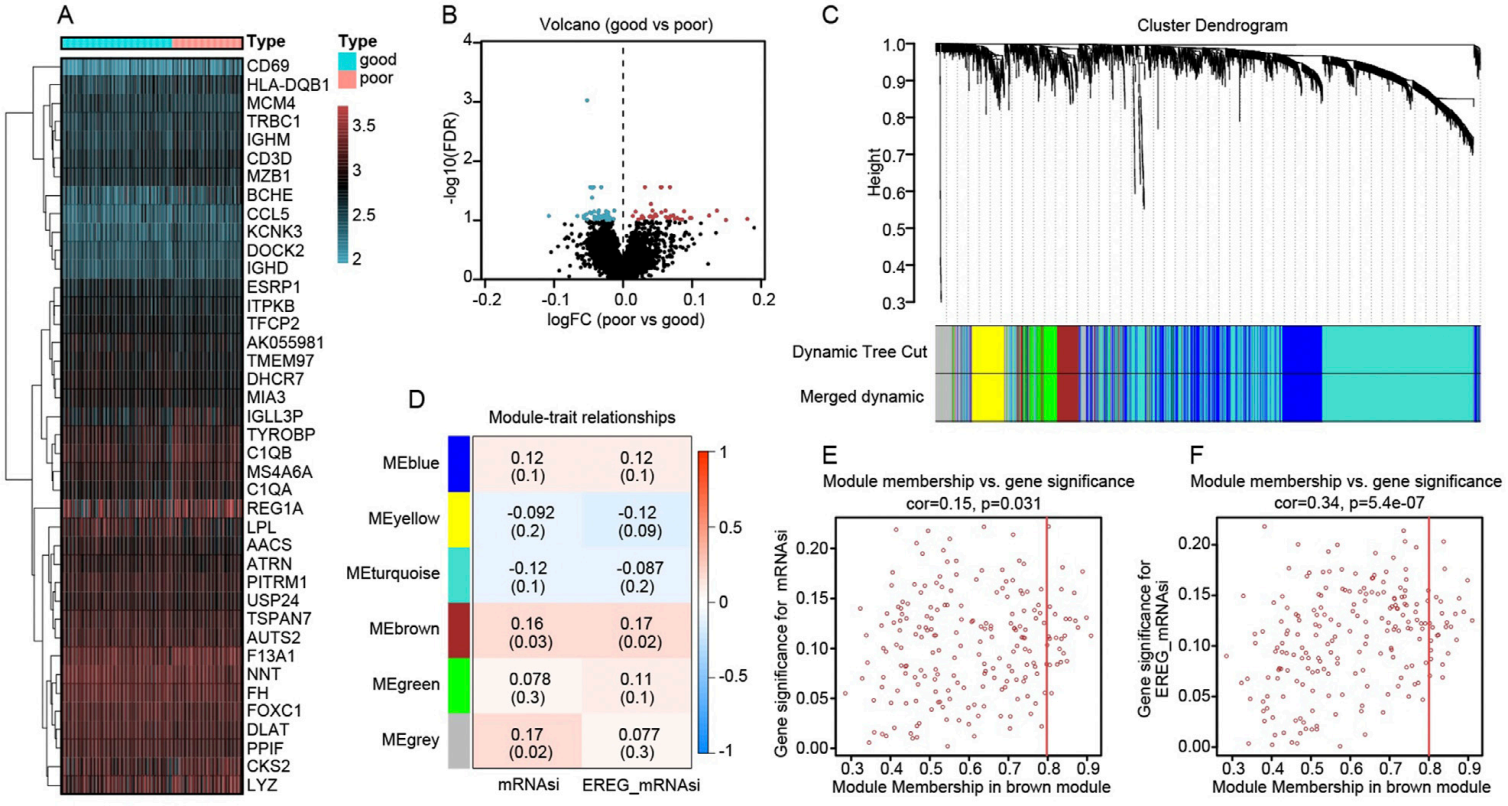
Differential expression and co-expression modules reveal key stemness signatures in kidney transplant recipients. **(A)** Heatmap of top differentially expressed genes comparing “good” (blue header) and “poor” (red header) renal outcome groups. Gene expression is represented by a gradient from low (blue/green) to high (red) values. **(B)** Volcano plot showing global distribution of differentially expressed genes. The x-axis indicates log fold change (poor vs. good), and the y-axis shows–log10(FDR). Points in blue denote genes upregulated in the good-outcome group, whereas red points denote genes overexpressed in the poor-outcome cohort. **(C)** Hierarchical clustering dendrogram of genes, with color-coded modules identified by dynamic tree cutting. The “merged dynamic” bar indicates the final module assignments. **(D)** Module–trait relationship heatmap, showing correlations (and p values) between each module and the mRNAsi or EREG_mRNAsi trait. Notably, the brown module is positively correlated with both indices. **(E)** Scatter plot relating gene significance for mRNAsi (y-axis) to module membership (x-axis) in the brown module (cor = 0.15, p = 0.031). **(F)** Scatter plot relating gene significance for EREG_mRNAsi (y-axis) to module membership (x-axis) in the brown module (cor = 0.34, p = 5.4e−07). The positive correlations imply that genes more central to the brown module also exhibit higher relevance to stemness traits.

We compared the transcriptomes of 192 kidney transplant recipients, dividing them into “good” versus “poor” graft-function groups based on post-transplant renal performance. A differential expression analysis identified a distinct set of genes significantly associated with clinical outcome. As illustrated in Figure 4A, the heatmap reveals clear clustering patterns: patients with good renal function (blue header) preferentially express certain immunoregulatory and B/T cell-related genes (e.g., *CD69*, *HLA-DQB1*, *MCM4*), whereas individuals with poor graft function (red header) exhibit higher expression of genes implicated in inflammation and metabolic stress (e.g., *CKS2*, *LYZ*). The corresponding volcano plot (Figure 4B) provides a global overview of these differences. The x-axis indicates the log fold change (logFC) in expression (poor vs. good), and the y-axis represents statistical significance [–log10(FDR)]. Genes depicted in blue circles on the left are more highly expressed in the “good” function group, whereas those in red circles on the right are upregulated in “poor” function samples. These divergent expression profiles suggest that graft outcomes may be influenced by distinct molecular pathways, highlighting potential targets for further functional validation and therapeutic intervention.

We applied Weighted Gene Co-expression Network Analysis (WGCNA) to the RNA-sequencing data of 192 kidney transplant recipients, using their mRNA stemness index (mRNAsi) and its epigenetically regulated variant (EREG_mRNAsi) as traits of interest. In Figure 4C, a hierarchical clustering dendrogram shows how genes were grouped into distinct modules based on co-expression patterns. The dynamic tree cut, and subsequent merging steps produced several color-labeled modules. Among these modules, the “brown” module displayed the strongest positive correlations with both mRNAsi and EREG_mRNAsi, as illustrated by the module–trait relationship heatmap in Figure 4D (correlation coefficients are shown in each cell, with corresponding *p* values in parentheses). To further explore the significance of this brown module, we examined the association between a gene’s module membership (its degree of connectivity within the module) and its “gene significance” (how closely it correlates with the stemness trait). The scatter plots in Figure 4E (for mRNAsi) and Figure 4F (for EREG_mRNAsi) demonstrate moderate yet statistically significant correlations, suggesting that genes highly connected within the brown module are also key contributors to stemness in the transplanted kidney context.

### Identification and functional characterization of eight key genes linked to graft outcomes

**FIGURE 5:**
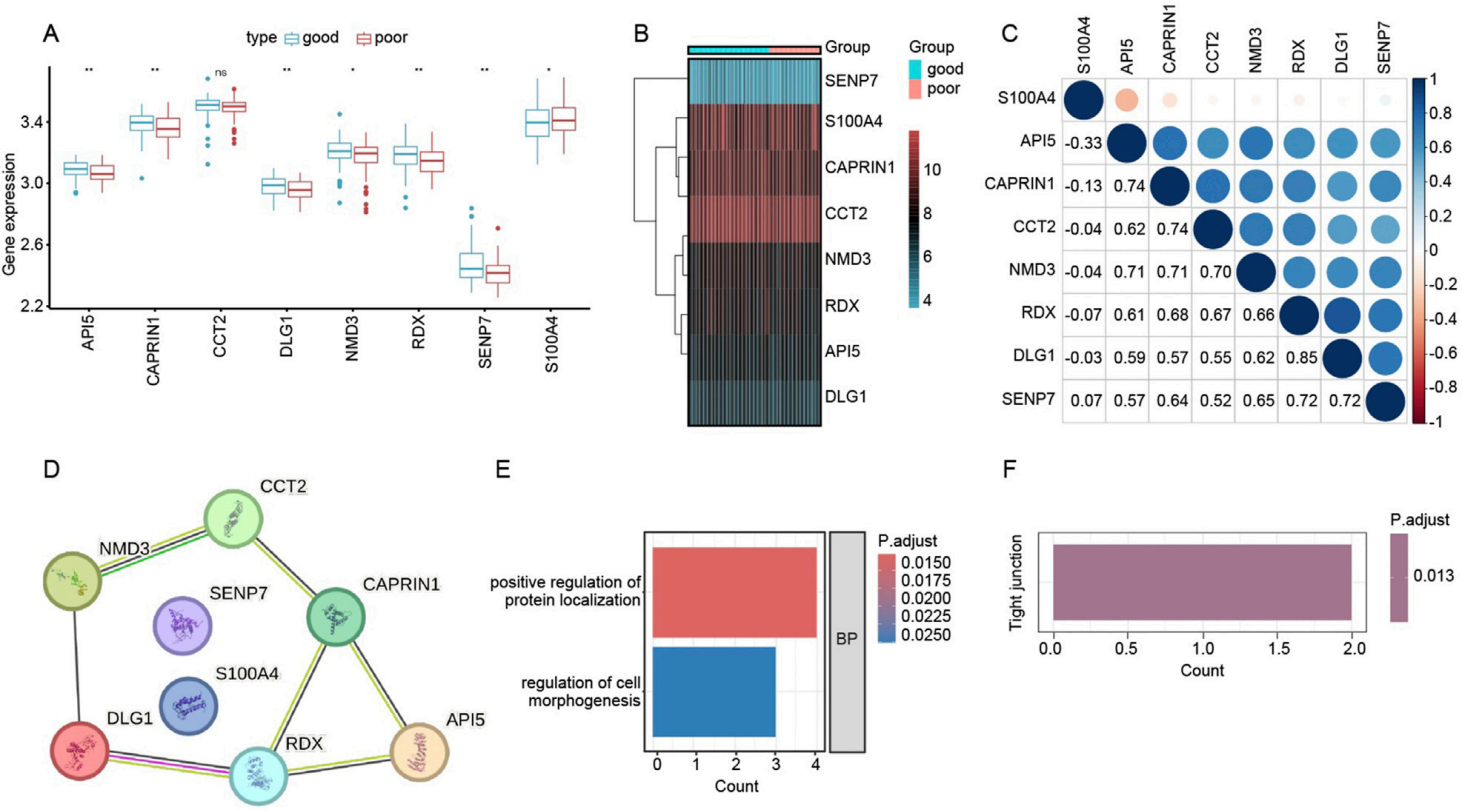
Functional analysis of key genes associated with renal allograft outcomes. **(A)** Box plots depicting log-transformed expression levels of the eight candidate genes (API5, CAPRIN1, CCT2, DLG1, NMD3, RDX, SENP7, and S100A4) in the “good” (blue) versus “poor” (red) outcome groups. Asterisks denote statistically significant differences (p < 0.05; p < 0.01). **(B)** Heatmap showing expression patterns of these eight genes across individual samples in each outcome group. Red coloring indicates higher expression, while blue/turquoise indicates lower expression. **(C)** Correlation matrix (Pearson’s r) illustrating pairwise relationships among the eight genes. Larger circles and deeper blues indicate stronger positive correlations, suggesting potential co-regulatory networks related to transplant outcomes. **(D)** STRING-derived PPI network for the eight genes. Nodes represent genes of interest, and edges reflect putative interactions or co-functional relationships. **(E)** GO Biological Process (BP) enrichment analysis showing top terms enriched among the eight genes, including “positive regulation of protein localization” (red bar) and “regulation of cell morphogenesis” (blue bar). The color scale indicates adjusted p-values. **(F)** KEGG pathway enrichment identifying “Tight junction” as a significantly associated pathway (p.adjust = 0.013), suggesting that these genes may impact cell junction integrity and possibly modulate graft survival.

To refine our search for critical genes underpinning renal allograft outcomes, we intersected the differentially expressed genes (DEGs) from the “good” vs. “poor” analysis with those constituting the WGCNA-derived brown module. This approach yielded eight key genes—*API5*, *CAPRIN1*, *CCT2*, *DLG1*, *NMD3*, *RDX*, *SENP7*, and *S100A4*. As illustrated in Figure 5A, box plots compare mRNA levels of the eight genes in patients with good outcomes (blue boxplots) versus poor outcomes (red boxplots). Several genes (*API5*, *CCT2*, *RDX*, and *S100A4*, among others) display statistically significant differences between the two groups, suggesting their potential roles in influencing transplant success or failure. In Figure 5B, a heatmap of these same genes highlights distinct expression profiles, with certain genes (e.g., *S100A4*) more prominently expressed in the poor-outcome cohort. Lastly, the correlation matrix in Figure 5C demonstrates that many of these genes exhibit moderate to strong positive correlations with one another, indicating possible co-regulation or shared pathways. Collectively, these findings pinpoint a small but potentially impactful gene set that may drive immune dysregulation or tissue injury in patients with suboptimal graft function.

We next examined potential functional interactions among the eight previously identified genes—*API5*, *CAPRIN1*, *CCT2*, *DLG1*, *NMD3*, *RDX*, *SENP7*, and *S100A4*—using the STRING database. As shown in Figure 5D, the Protein–Protein Interaction (PPI) network suggests multiple direct or closely related connections among these candidates, indicating that they may participate in shared pathways relevant to graft outcome.

To clarify their biological functions, we performed Gene Ontology (GO) and KEGG pathway enrichment based on these eight genes. Figure 5E highlights two principal GO Biological Process (BP) terms: “positive regulation of protein localization” and “regulation of cell morphogenesis.” Meanwhile, Figure 5F (not shown in full here) indicates significant enrichment in the “Tight junction” pathway (p.adjust = 0.013), hinting that these genes could influence cell–cell junction integrity and tissue remodeling in the context of kidney transplantation. Collectively, these findings propose novel mechanistic links by which these eight genes may modulate immune or structural dynamics in the transplanted kidney.

## Discussion

Our single-cell analysis highlights a dysregulated immune microenvironment in patients with impaired graft function, characterized by pro-inflammatory T cell subsets and innate immune activation. These findings align with broader literature emphasizing that transplant outcomes hinge on the balance between regulatory and effector immune mechanisms (Hanidziar and Koulmanda, 2010). Notably, we observed a skewing toward Th17 responses over regulatory Tregs in dysfunctional grafts. Such Th17/Treg imbalances are well-documented drivers of rejection: Th17 cells can mediate allograft damage and are relatively resistant to suppression by Tregs (Benghiat et al., 2008; Heidt et al., 2010). Furthermore, inflammatory cytokine milieus not only favor Th17 differentiation but can also destabilize the Treg lineage–in effect converting established Tregs into Th17-like cells under pro-inflammatory conditions (Chung et al., 2009; Wei et al., 2007). This plasticity is consistent with prior reports that adverse graft inflammation creates resistance to tolerance induction by preventing Treg development and function (Ciofani et al., 2012). In summary, our data reinforce the paradigm that unchecked inflammation tilts the immune equilibrium towards graft-destructive pathways, echoing the broader consensus that controlling intragraft inflammation and fostering Treg dominance are key to promoting tolerance (Hang et al., 2019; Miossec and Kolls, 2012). Importantly, our study extends these concepts by providing granular, single-cell evidence of Th17/Treg disruption in human transplant recipients, thereby linking classical immune regulation paradigms to specific cellular and molecular players in the failing graft.

A novel aspect of our study is the application of transcriptional stemness indices (mRNAsi and EREG-mRNAsi) to immune cells in the transplant setting. The mRNA expression-based stemness index (mRNAsi), originally developed in oncology as a measure of how closely a cell’s transcriptome mirrors that of stem cells (Malta et al., 2018; Molinier-Frenkel et al., 2019; Nabors et al., 2017), was repurposed here to gauge T cell differentiation states. Although the mRNA-based stemness index (mRNAsi) and its epigenetically weighted derivative EREG_mRNAsi were first described in oncology, the underlying one-class logistic regression (OCLR) model was actually trained to discriminate pluripotent (ESC/iPSC) from fully differentiated adult tissues. Thus, the score captures generic transcriptional programs that endow cells with self-renewal and multipotency and has proven informative across more than 30 normal tissues and developmental stages (Malta et al., 2018). Building on this conceptual breadth, subsequent studies have successfully repurposed mRNAsi in immune settings—for instance, an integrative analysis of colorectal cancer showed that higher mRNAsi values were strongly associated with activated memory CD4^+^ T cells, underscoring the index’s ability to reflect “stem-like” T-cell states (Ye et al., 2022). High mRNAsi scores in graft-infiltrating T cells suggest a less-differentiated, self-renewing phenotype–analogous to T memory stem cells–whereas lower scores indicate terminally differentiated effectors. Clinically, this finding raises intriguing implications: a pool of long-lived, stem-like T cells could perpetuate chronic alloimmune responses despite conventional immunosuppression. This is reminiscent of observations in chronic infections and cancers where a subset of progenitor-exhausted T cells sustains the immune reaction over time (the “stemness” of T cells) (Goronzy and Weyand, 2017; Graef et al., 2014; Kallies et al., 2020). In transplantation, such cells might continually refuel anti-graft reactivity or, conversely, could be harnessed for tolerance if steered toward a regulatory fate. The plasticity of immune cells is a double-edged sword: while stem-like T cells provide adaptability and longevity to immune responses, their fate can be redirected by the microenvironment. For instance, as noted above, pro-inflammatory signals may redirect tolerogenic Tregs into pathogenic Th17 cells (Chen and Wood, 2007; Koenders and van den Berg, 2010; Zheng et al., 2008). Our pseudotime trajectory analysis indeed suggested fluid transitions along the Th17–Treg axis, underscoring that immune phenotypes in the graft are not fixed. From a clinical standpoint, these results underscore the need to modulate the local cytokine milieu–for example, by blocking inflammatory cues (IL-6, IL-1β) – to prevent harmful lineage switching and to possibly maintain a reservoir of exhaustion or regulation-prone T cells. In sum, integrating stemness indices with T cell biology provides a framework to understand how certain T cells in a transplant might acquire a self-renewing, plastic state that can either hinder or help graft tolerance depending on how it is therapeutically manipulated.

All participants in this study received calcineurin-inhibitor (CNI)–based immunosuppression (e.g., tacrolimus or cyclosporin A). Because CNIs inhibit NFAT signaling and thereby affect T-cell activation, metabolism, and lineage commitment, prolonged exposure could alter the maintenance of T-cell stemness and the Th17/Treg balance. Drug effects may therefore interact with transplant-related immune status and confound the transcriptomic features observed here. Our cohort lacks systematic trough-level monitoring and detailed pharmacokinetic data, so we could not fully adjust for this factor at the single-cell level. Future work will: (i) record individual CNI dosages and concomitant blood levels in an expanded cohort, sampling them in parallel with immune profiling; (ii) recruit recipients maintained on alternative regimens such as mTOR inhibitors or belatacept for comparative analysis; and (iii) treat PBMCs *in vitro* with graded CNI concentrations to assess direct effects on T-cell stemness markers and Th17 differentiation. These strategies should clarify how immunosuppressants modulate T-cell lineage fate and provide a foundation for fine-tuning immunotherapy in transplant recipients.

Our findings also shed light on the intercellular communication networks driving graft inflammation. Using the iTalk ligand–receptor modeling, we identified robust crosstalk between innate and adaptive immune compartments. A striking example is the S100 family protein-mediated signaling: S100 transcripts (particularly S100A4) were highly expressed by graft-infiltrating myeloid cells, with predicted engagement of Toll-like receptor 4 (TLR4) on neutrophils and other innate immune cells. This mechanism is supported by the literature, as many S100 proteins act as extracellular alarmins that bind pattern recognition receptors like TLR4 and RAGE to amplify inflammation (Donato, 2001). S100A4 is known to bind TLR4, triggering NF-κB signaling and cytokine release (Abdelfattah et al., 2022; Wu et al., 2021). Thus, our data suggest a feed-forward loop wherein S100A4 released in the graft can recruit and activate neutrophils via TLR4, exacerbating tissue damage. This is in line with recent reports that blocking S100A4 function markedly dampens neutrophil infiltration in inflamed tissues (Chen et al., 2015; Sun et al., 2021). Moreover, S100A4 and related S100 alarmins have been correlated with elevated neutrophil-to-lymphocyte ratios and systemic inflammation (Bagheri-Hosseinabadi et al., 2022; Liu et al., 2021), a pattern mirrored in our patients with poor graft function. Beyond S100A4, the intercellular communication analysis highlighted other critical cytokine and chemokine interactions: for example, high expression of IL-6 and IL-1β in myeloid clusters (upstream drivers of Th17 differentiation) and potential T cell to myeloid signals like GM-CSF or IFN-γ that could further activate macrophages (Kleinewietfeld et al., 2013; Wang W. et al., 2018). These interactive networks emphasize that graft rejection is not solely a T cell-autonomous process, but rather a concerted dialog between innate and adaptive cells. Neutrophil recruitment emerges as a notable feature of impaired grafts, likely orchestrated by damage-associated signals such as S100-TLR4 interactions and chemokines. Clinically, this insight points to the potential benefit of interrupting these dangerous liaisons–for instance, TLR4 antagonism or S100 neutralization might reduce the innate immune-driven inflammatory amplification within the graft. Overall, the intercellular crosstalk analysis reinforces that effective control of rejection may require targeting these upstream innate triggers and not just T cells alone.

Weighted gene co-expression network analysis (WGCNA) yielded eight hub genes (API5, CAPRIN1, CCT2, DLG1, NMD3, RDX, SENP7, S100A4) associated with the stemness and dysfunction signatures in our cohort. These genes, though not classical immune markers, appear to be pivotal intracellular regulators that modulate immune cell survival, activation, and interaction. API5 (Apoptosis Inhibitor 5), for instance, is an anti-apoptotic factor that can be secreted and function as a DAMP; it has been shown to bind TLR4 on dendritic cells and stimulate NF-κB activation (Kim et al., 2018). Elevated API5 in rejecting grafts could thus promote dendritic cell activation and prolong T cell survival, fueling chronic rejection. CAPRIN1 is an RNA-binding protein integral to cell cycle progression; its absence impairs proliferation of immune cells (Wang et al., 2005), indicating CAPRIN1 supports the expansion of allo-reactive lymphocytes. CCT2, a subunit of the CCT (TRiC) chaperonin complex, is essential for folding cytoskeletal proteins (actin, tubulin) during T cell activation (Andres-Delgado et al., 2012; Martin-Cofreces et al., 2021). Upregulation of CCT2 likely reflects vigorous T cell activation and proliferation in failing grafts, as properly folded cytoskeletal elements are needed for immunological synapse formation and clonal expansion. Another scaffold is DLG1 (Discs Large Homolog 1), which organizes TCR signaling microclusters; DLG1 promotes the assembly of kinase complexes at the T cell–APC interface and its loss disrupts early TCR signaling 34960191. The enrichment of DLG1 in our data suggests a state of heightened TCR engagement, and by extension, that targeting downstream DLG1-interacting pathways might modulate T cell activation intensity.

The other hub genes further underscore the cellular metabolic and migratory machinery driving graft immunopathology. NMD3 is a nuclear export adaptor for the 60S ribosomal subunit (Sengupta et al., 2010), required for robust protein synthesis. High NMD3 expression implies increased ribosome biogenesis, consistent with rapidly proliferating immune cells in an active rejection milieu. RDX (Radixin), a member of the ezrin-radixin-moesin family, links actin filaments to the plasma membrane. ERM proteins like radixin are crucial for lymphocyte morphology changes during migration and immune synapse stabilization (Pore and Gupta, 2015). Radixin upregulation may facilitate the migration of effector cells into the graft and stable contact with target cells (e.g., graft endothelial cells or APCs). SENP7, a SUMO-deconjugating protease, has recently been shown to sustain CD8^+^ T cell metabolic fitness by removing SUMO modifications from metabolic regulators (such as PTEN) under oxidative stress (Wu et al., 2022). Thus, SENP7 in graft-infiltrating T cells could enhance their glycolytic and survival capacity in the inflamed, nutrient-poor graft environment, thereby prolonging their effector functions. Lastly, S100A4 – already discussed as an extracellular factor–may also have intracellular roles in immune cells, but its primary significance here is as a secreted pro-inflammatory mediator connecting this gene network to tissue inflammation. Collectively, these eight genes represent key nodal points in immune cell survival (API5, SENP7), proliferation and protein synthesis (CAPRIN1, NMD3), activation (DLG1, CCT2), migration (RDX), and inflammatory signaling (S100A4). Each presents a potential therapeutic angle: for example, blocking S100A4-TLR4 interactions to reduce neutrophil recruitment, or inhibiting API5 to limit unwanted dendritic cell activation. While targeting core cellular processes like CCT2 or NMD3 is less specific and could be toxic, their identification highlights the intense metabolic and biosynthetic demands of anti-graft immune cells, suggesting that metabolic interference (e.g., mTOR inhibition) might preferentially affect these highly active cells. Future therapeutics might also explore SENP7 or related metabolic checkpoints to preferentially impair effector T cells’ fitness while sparing regulatory cells. In summary, the hub genes not only provide mechanistic insight into the cellular state of rejection but also reveal non-canonical targets (like S100A4 and API5) that warrant further investigation for immune modulation.

This study’s strength lies in its multi-dimensional single-cell approach, enabling an unprecedented resolution of the immune landscape in kidney transplant patients with graft dysfunction. By employing single-cell transcriptomics, we captured the heterogeneity of immune infiltrates that bulk assays would obscure, distinguishing subtle states of T cells (e.g., putative stem-like vs. terminal effector) and myeloid cells (e.g., pro-inflammatory neutrophils, monocyte-derived dendritic cells, etc.). The addition of pseudotime trajectory analysis allowed us to reconstruct dynamic differentiation pathways *in silico*, providing clues about how, for example, a Treg might diverge toward a Th17 phenotype under inflammatory pressure. This temporal modeling strengthens the causal interpretations of our snapshot data by suggesting possible lineage relationships and progression patterns of immune cells. Another notable strength is the use of intercellular communication modeling (iTalk) to integrate information across cell types. Rather than analyzing T cells or myeloid cells in isolation, iTalk helped identify critical ligand–receptor pairs (such as S100A4–TLR4, IL-6–IL-6R, CXCL chemokines–CXCR2 on neutrophils) that drive the coordination of rejection. This systems-level view mirrors the complex cell–cell interactions *in vivo* and generates testable hypotheses about interrupting these signals. Lastly, our WGCNA-based gene discovery pipeline provided a data-driven means to pinpoint key regulatory genes within the co-expression network correlated with clinically relevant traits (stemness index and graft function). This unbiased approach led us to genes like API5 and SENP7 that might have been overlooked by conventional differential expression analyses. Integrating these advanced methodologies–single-cell RNA-seq, pseudotime modeling, ligand–receptor analysis, and network-based gene selection–is an innovative aspect of our work, yielding a comprehensive picture from molecular regulators to intercellular interactions. We believe this integrative strategy is a blueprint for dissecting complex immunological diseases beyond transplantation as well.

Validating our key molecular interactions remains a priority. Determining S100A4 levels in tissue or blood across independent cohorts and correlating expression with neutrophil infiltration and graft function would lend credibility to the single-cell transcriptomic findings. Model systems, such as mouse transplant models or *ex vivo* human assays, could confirm whether blocking S100A4–TLR4 alleviates neutrophil-driven tissue injury. Moreover, IL-6 or IL-1β blockade might tip the Th17/Treg balance toward tolerance, enhancing graft survival. Another compelling avenue is inducing T cell exhaustion or senescence to limit the persistence of high-stemness T cells, while bolstering Treg stability (e.g., via IL-2 receptor agonists or epigenetic modulators) to prevent Th17 conversion under inflammatory conditions.

Longitudinal studies tracking immune phenotypes from pre-to post-transplant could reveal whether mRNAsi shifts or rising API5 and S100A4 levels foreshadow rejection, enabling earlier therapeutic intervention. Parallel integration of spatial transcriptomics or multiplex imaging with our single-cell data would clarify the microanatomic context of inflammatory lesions, such as neutrophil clustering around S100A4-secreting cells. Ultimately, translating these molecular insights into biomarkers or treatments can guide more precise, tolerance-promoting strategies for transplant recipients, advancing beyond our current snapshot to a dynamic, patient-tailored approach in managing graft function. Although this study used iTalk to systematically predict ligand-receptor interactions from single-cell transcriptomic data, these *in silico* inferences have not yet been functionally validated. Whether the predicted pairs truly drive signal exchange between peripheral T cells and myeloid cells in transplant recipients remains to be confirmed. Going forward, we will establish homologous and heterologous co-culture systems and apply neutralizing antibodies against S100A4/S100A8/A9 or TLR4 inhibitors to evaluate the direct role of the S100–TLR4 axis in Th17 expansion, maintenance of T-cell stemness, and amplification of inflammation. In parallel, we will employ CRISPR-Cas9 knockouts or siRNA-mediated knockdowns of key ligands/receptors, together with phospho-NF-κB reporter assays, to rigorously verify the biological relevance of our computational predictions. These follow-up experiments are expected to clarify the functional significance of the inferred signaling pathways in transplant-related immune dysregulation and lay the groundwork for novel precision immunotherapeutic strategies. To ensure sufficient cell numbers while controlling sequencing costs, we pooled peripheral blood mononuclear cells (PBMCs) from 10 participants per group before sequencing. However, without cell hashing or individual sample barcoding, we could not trace each cell back to its donor, preventing assessment of inter-individual biological variability. This limitation reduces the granularity of our analyses and may affect the generalizability of our conclusions. In future studies, we will incorporate multi-tag strategies—such as oligonucleotide-based cell hashing, MULTI-seq, or CITE-seq—to retain donor-level provenance while remaining cost-effective. We will also apply deconvolution algorithms like Demuxlet to disentangle mixed-sample data, enabling systematic comparison of immune heterogeneity across individuals and enhancing the external validity of our findings. This study employed a cross-sectional design, with peripheral blood sampled only once for transcriptomic and proteomic profiling. Consequently, we could not track real-time changes in T-cell stemness and inflammatory status after transplantation, nor determine causal links between these immune states and graft function over time. The absence of longitudinal follow-up limits the reconstruction of immune trajectories and may obscure early warning signals. In future work, we plan to establish a multi-time-point cohort, collecting serial samples at 1, 3, 6, and 12 months post-transplantation and during any clinically confirmed rejection episodes. By integrating barcoded single-cell sequencing, flow cytometry, and Bayesian dynamic modeling, we aim to systematically delineate the temporal features of T-cell stemness exhaustion and inflammatory imbalance during graft adaptation or rejection, thereby defining optimal windows for precision intervention. Our study used a single-time-point transcriptomic snapshot to perform pseudotime modeling and infer differentiation trajectories—such as the transition from CD4^+^ T cells to Th17 cells. It is important to note that pseudotime algorithms reconstruct a “pseudo-temporal” order from static expression differences; they lack true temporal resolution and lineage-tracing markers, making it impossible to definitively assign directionality or rule out parallel differentiation paths. Although we added RNA velocity and shared TCR-clonotype analyses to partially support the inferred trajectory, these approaches cannot replace longitudinal sampling or experimental lineage tracing. Future work will: (i) integrate barcoded scRNA-seq, scTCR-seq, and RNA velocity on serial follow-up samples to enhance temporal information; (ii) employ lineage-tracing techniques driven by mitochondrial mutations or CRISPR barcodes, along with *in-vitro* differentiation and adoptive transfer into humanized mice, to empirically validate key differentiation nodes; (iii) combine multi-omics (scATAC-seq, CITE-seq) to map epigenetic and protein-level dynamics, thereby clarifying T-cell fate decisions comprehensively.

In conclusion, this single-cell study illuminates how reduced T-cell stemness, coupled with S100-driven neutrophil recruitment, contributes to graft dysfunction in kidney transplant recipients. Our data underscore a Th17/Treg imbalance, heightened inflammatory signals, and the involvement of eight key genes modulating immune activation and tissue remodeling. Such insights emphasize the need for interventions that restore T-cell plasticity and curb pathogenic Th17 programs, potentially by targeting S100–TLR4 axes or metabolic checkpoints. Future longitudinal investigations may validate these findings, guide personalized immunosuppressive strategies, and ultimately improve allograft outcomes through earlier detection and targeted modulation of immune dysregulation.

## Data Availability

The datasets presented in this study can be found in online repositories. The names of the repository/repositories and accession number(s) can be found below: https://www.ncbi.nlm.nih.gov/geo/query/acc.cgi?acc=GSE298312.

## Supplementary material

SUPPLEMENTARY FIGURE S1 Elevated plasma IL-17A in kidney-transplant recipients with a high-Th17 CD4^+^ T-cell transcriptional profile **(A)** Representative immunoblots for S100A4 (37 kDa) and the loading control β-actin (43 kDa) in peripheral-blood mononuclear cells from three kidney-transplant recipients with graft dysfunction (PTRI, lanes P1–P3) and three with stable graft function (CTRL, lanes P1–P3); “P” denotes individual patients. **(B)** Bar chart summarising densitometric quantification of S100A4 normalised to β-actin (mean ± SD, *n* = 3 patients per group). * indicates *P* < 0.01 by two-tailed Wilcoxon test, confirming a ∼2.5-fold increase in the PTRI cohort. **(C)** Box-and-jitter plot of plasma IL-17A concentrations measured by Luminex multiplex assay in recipients whose graft biopsies exhibited a poor graft function (n = 7) versus recipients with a stable graft function (n = 8). Boxes show the inter-quartile range; horizontal lines, medians; whiskers, 1.5 × IQR; dots, individual patients. Significance was assessed with a two-sided Mann–Whitney U test.
