# Correction: Reduced T-cell stemness underlies Th17 expansion and graft dysfunction in kidney transplant recipients

**DOI:** 10.3389/fgene.2025.1658563

**Published:** 2025-07-15

**Authors:** Chang Liu, Hao Jiang, Andu Zhu, Chen Xu, Zhenfan Wang, Guocai Mao, Minjun Jiang, Jianchun Chen, Zheng Ma, Jiaqian Qi, Zhijun Cao

**Affiliations:** ^1^Department of Urology, Suzhou Ninth People’s Hospital, Soochow University, Suzhou, China; ^2^Department of Urology, The First Affiliated Hospital of Soochow University, Suzhou, China; ^3^Department of Clinical Laboratory, Suzhou Ninth People’s Hospital, Soochow University, Suzhou, China; ^4^Department of Thoracic Surgery, Suzhou Dushu Lake Hospital, Dushu Lake Hospital Affiliated to Soochow University, Medical Centre of Soochow University, Suzhou, China; ^5^Department of Thoracic Surgery, The First Affiliated Hospital of Soochow University, Soochow University, Suzhou, Jiangsu, China; ^6^Department of Hematology, The First Affiliated Hospital of Soochow University, Suzhou, China

**Keywords:** T-cell stemness, Th17 cells, S100 proteins, kidney transplantation, immune regulation

The **Data availability statement** was erroneously given as “The datasets presented in this study can be found in online repositories. The names of the repository/repositories and accession number(s) can be found below: https://www.ncbi.nlm.nih.gov/geo/query/acc.cgi?acc=GSE276822.” The correct **Data availability statement** is “The datasets presented in this study can be found in online repositories. The names of the repository/repositories and accession number(s) can be found below: https://www.ncbi.nlm.nih.gov/geo/query/acc.cgi?acc=GSE298312.”

The authors apologize for this error and state that this does not change the scientific conclusions of the article in any way. The original article has been updated.

