# Supplementary figures and images for: Reduced T-Cell stemness underlies Th17 expansion and graft dysfunction in kidney transplant recipients
Source: Front Genet. 2025 Jun 13;16:1588941. doi: 10.3389/fgene.2025.1588941 (PMC12202220; doi:10.3389/fgene.2025.1588941)

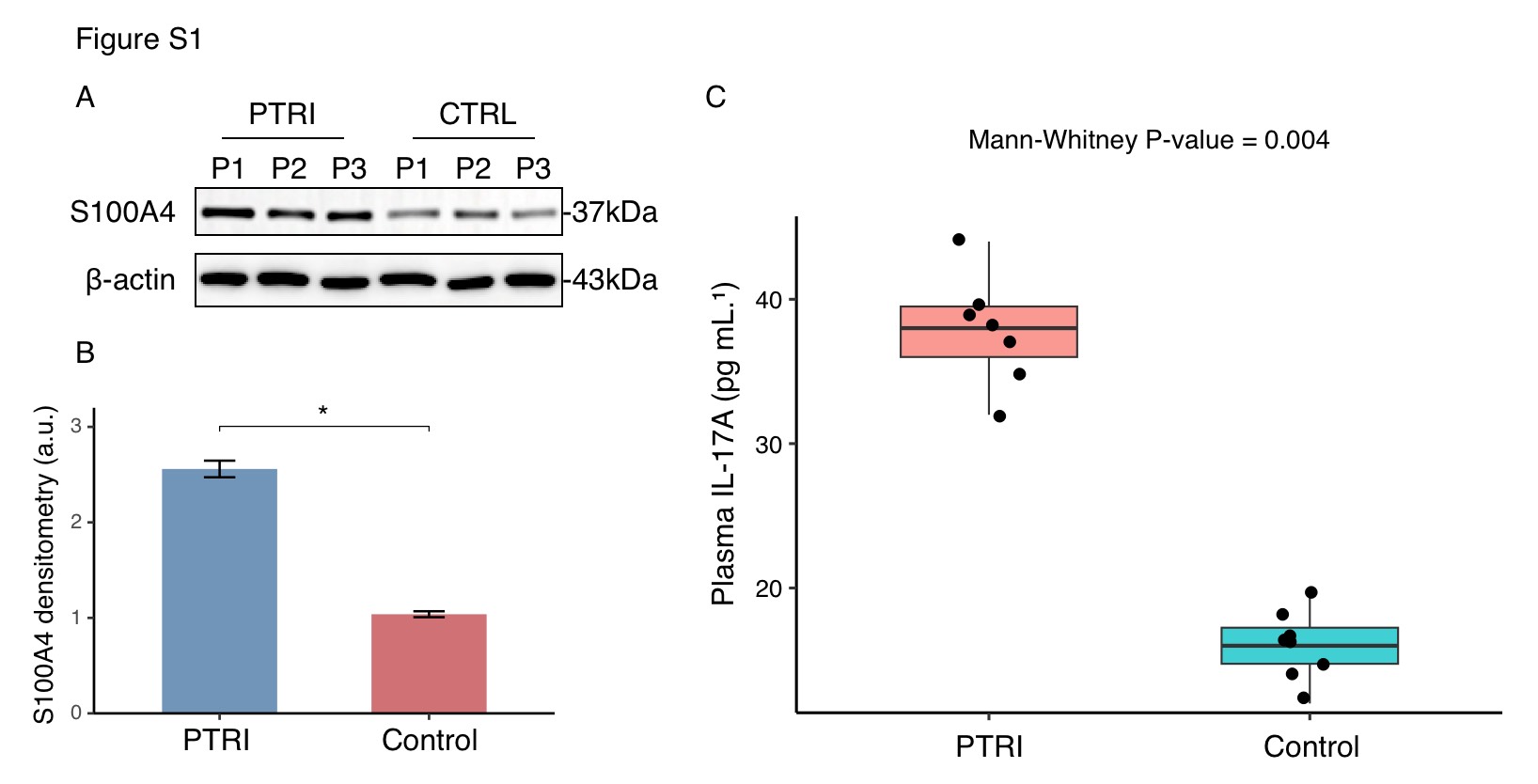

Supplement: Supplementary file 1 [file Image1.jpeg]
