# Supplementary material for: Reduced T-Cell stemness underlies Th17 expansion and graft dysfunction in kidney transplant recipients
Source: Front Genet. 2025 Jun 13;16:1588941. doi: 10.3389/fgene.2025.1588941 (PMC12202220; doi:10.3389/fgene.2025.1588941)
Supplement: Supplementary file 2 [file DataSheet1.zip › rawdatas/Flowchart Description(worksheet).docx]

**Flowchart Description**

1. **Patient Enrollment & Grouping**

• Recruit 20 adult kidney transplant recipients: 10 with stable graft function and 10 with impaired graft function.

• Record demographic/clinical data and obtain written informed consent.

2. **Blood Sampling & PBMC Isolation**

• Collect ~10 mL peripheral blood from each participant.

• Isolate PBMCs by Ficoll–Hypaque density gradient centrifugation.

• Assess cell viability (>90%) via trypan blue exclusion.

3. **Sample Pooling & scRNA-Seq Library Preparation**

• Pool equal numbers of PBMCs from each subject in the same outcome group to form two composite samples (Control vs. Renal Insufficiency).

• Load each pooled sample onto a 10x Genomics Chromium Controller.

• Perform cDNA synthesis, library construction, and quality checks.

• Sequence libraries on an Illumina platform (2×150 bp reads, ~50,000 reads/cell).

4. **scRNA-Seq Data Processing & Quality Control**

• Demultiplex and align reads to the GRCh38 reference using Cell Ranger.

• Filter out low-quality cells (fewer than 200 genes or >15% mitochondrial content) and potential doublets.

• Normalize gene expression for downstream analyses.

5. **Clustering & Cell-Type Annotation**

• Identify highly variable genes and perform dimensionality reduction (PCA, UMAP).

• Cluster cells (Louvain algorithm), then annotate clusters based on canonical marker genes (e.g., CD3D, LYZ).

6. **Key Analyses**

• **Differential Expression & Pathway Enrichment**: Compare expression patterns across clusters and between outcome groups.

• **Pseudotime Trajectories (Monocle)**: Reconstruct lineage paths of CD4⁺ T cells, Th17 cells, and Tregs to investigate developmental branching and S100A4 expression.

• **Ligand–Receptor Communication (iTalk)**: Identify intercellular signaling pathways among major immune subsets (e.g., S100–TLR4).

• **Stemness Indices (mRNAsi & EREG_mRNAsi)**: Quantify stem-like transcriptional profiles in T cells.

7. **Validation with Bulk RNA-Seq (GEO Dataset)**

• Obtain an independent cohort (n=192) from GEO.

• Calculate mRNAsi/EREG_mRNAsi and correlate with clinical outcomes.

• Perform WGCNA to link co-expression modules with stemness scores and graft function.

8. **Identification of Key Genes & Functional Insights**

• Intersect differentially expressed genes with WGCNA “brown” module genes.

• Highlight 8 hub genes (e.g., S100A4, API5) potentially driving immune dysregulation.

• Conduct functional enrichment (GO/KEGG) to interpret roles in immune homeostasis and graft injury.

9. **Biological Interpretation & Conclusions**

• Summarize that diminished T-cell stemness correlates with a Th17 shift and S100 protein–mediated neutrophil recruitment, driving graft dysfunction.

• Propose potential therapeutic strategies targeting these molecular networks to enhance graft tolerance and long-term transplant survival.
